# Consumers’ Perceived Value of Pork Meat: A Segmentation on Intrinsic and Extrinsic Cues

**DOI:** 10.3390/foods14132324

**Published:** 2025-06-30

**Authors:** Diewald Jordaan, Annchen Mielmann, Carike Brits

**Affiliations:** The Africa Unit for Transdisciplinary Health Research (AUTHeR), North-West University, Potchefstroom 2531, South Africa

**Keywords:** perceived value, pork meat, intrinsic cues, extrinsic cues, segmentation

## Abstract

This study aimed to determine the intrinsic and extrinsic product cues affecting consumers’ perceived value of pork meat. A segmentation study of seventeen intrinsic (*n* = 103) and twenty-six extrinsic cues (*n* = 114) on a South African sample was implemented. Cluster analyses provided two consumer segments for the product cues: the sensory seekers and the indecisive consumers for the intrinsic cues, and the price seekers and the preparation inquirers for the extrinsic cues. The sensory seekers valued the visual appearance, odour, taste, and flavour of pork as moderately to very important. The preparation inquirers regarded all the extrinsic cues, except for the processing cues, as moderately to very important. Accentuating the importance of sensory and preparation cues in pork meat products may contribute to improving the quality of products. This paper highlights that more research is needed on how consumers could benefit from the cue-adjusted pork meat products that will influence their perceived value of this affordable and versatile food in the South African context.

## 1. Introduction

Globally, pork is the most consumed red meat [1]. Pork consumption has increased globally by 77% from 63.5 million tons in 1990 to 113 million tons in 2022. With the world population that has already surpassed the 8 billion mark, this trend in increasing pork consumption will continue, requiring an increased supply of pork. [2] In South Africa between 2015 and 2024, pork saw the largest growth in total domestic consumption (38.5%—or nearly 90 thousand tons) while beef (0.9%—equivalent to 7 thousand tons) and poultry (1.8%—equivalent to 35.8 thousand tons) grew marginally, and sheep meat consumption declined by 27.6% (37.4 thousand tons) [3]. In South Africa, the pork industry has grown tremendously as it contributes 10.44% to the total South African manufactured meat and meat products. From 2021, the production of pork meat and pork meat products increased to a total quantity of over 139 thousand tonnes, equivalent to more than R6.7 billion [4].

Pork meat is a versatile source of protein as it is suitable for almost any dish or meal due to the variety of primal cuts and value-added products that are available on the market, for example, breakfast meats like bacon and pork bangers, deli meats like sandwich ham, and sausages, as well as primal cuts ideal for dinners like belly ribs and pork fillets [5,6]. Pork meat continues to provide beneficial value to consumers through essential macro- and micronutrients [7,8].

Pork is mainly sold in South Africa via two separate chains: fresh pork and processed pork products [9]. Fresh pork mainly consists of primal cuts, which do not undergo any further processing steps, while processed pork product consists of manufacturing processes and techniques to create an altered and enhanced product [10]. Primal cuts, which are the large unprocessed limbs or muscles of the animal, are either sold as sub-primal cuts or sent for further processing. Processed pork products are primal cuts that are transformed and utilised differently depending on the product outcome [11]. Processed pork products are generally ready-to-eat [12]; however, most of the raw material undergoes processing, which develops improved taste, visual appearance, texture, and preservation [13]. Processing meat involves several different methods, such as fermenting, salting, curing, and smoking, for the purpose of enhancing sensory qualities and shelf life [12].

The wide range of pork meat products makes it more accessible to a large contingent of consumers than other red meats [14], as consumers have more options to choose from that appeal to their demographic segmentation and behavioural characteristics, such as purchasing power and consumption habits [15]. Consumers’ behavioural characteristics can be shaped further by product cues.

Cues are signals representing the development context and attributes of things. In a sense, a cue is a collection of information. This kind of cue or collection of cues will influence the judgment and decision-making of individuals [16]. The distinctive product cues of pork meat, such as colour, protein content, and price per kilogram, set it apart from other red meat commodities. Consumers analyse intrinsic and extrinsic product cues as indicators depicting the quality of the product [17,18]. Quality is important to consumers as it is created from their own perception of combinations of different product attributes such as appearance, price, and ingredients in order to process purchasing decisions [19,20]. Furthermore, food quality is a multi-dimensional notion that is impacted by contextual elements since consumer quality evaluation is a relevant and personal process that affects their purchasing decisions [20].

Intrinsic product cues consist of product-related attributes and tangible attributes of the product, such as its flavour, texture, appearance, aroma, and nutritional value, which are not influenceable without modifying the product’s biological properties [21]. These attributes serve as the fundamental benchmark by which consumers assess the value of pork meat since they are closely linked to its physical characteristics and quality. The factors influencing the intrinsic cues of pork meat on consumers’ purchasing intention include product quality, product features and functionality, perceived risk and uncertainty, and brand reputation and heritage.

In comparison, extrinsic cues are outside characteristics that are not directly related to the product itself but have a significant impact on how consumers perceive it [22], which include packaging, processing, quality, additives, price, and place of origin [23]. These extrinsic cues frequently function as proxies for ethical, safety, and quality reflections, whereby consumers’ perceived value of pork meat is shaped. The factors influencing the extrinsic cues of pork meat on consumers’ purchasing intention include branding, labelling, in-store display, and marketing. These cues influence each other as they battle with the customers’ existing perceived product value and the newly obtained product information. Consumers utilise extrinsic cues to determine product quality in settings where the intrinsic aspect of the products is not accessible for evaluation [24]. However, the intrinsic product cues become the key factor in the re-purchasing intention process as the expected quality through extrinsic cues is compared and evaluated to formulate consumers’ perceived value of the product [25,26].

Both intrinsic and extrinsic cues can be directed toward generating positive sensory expectations, which can then dictate judgments of experienced quality upon consumption [5]. Sight is usually the first sense connected to our evaluation of foods [6]. While taste is reported to have the greatest influence on food choice [7], visual information is typically processed prior to food entering the mouth [6] and can even influence flavour perception [14]. These visual cues are not limited to intrinsic characteristics of the product itself (e.g., portion size, shape, colour) but also pertain to extrinsic characteristics such as food packaging [6]. In addition to packaging design (e.g., colour scheme, transparency/opacity, pictures), written information presented thereupon can also affect perceived value and expected outcomes, including how the product will be liked [15,16]. Ultimately, visual cues can be very impactful and influence consumer sensory expectations, hedonic evaluations, and emotions associated with food products and the overall eating experience [17,18,19,20,21].

The demand for high-quality pork meat products has increased, as consumers are becoming more concerned about the quality, safety, and environmental impact of food. Based on the proposition that consumers’ perceived value can be markedly influenced by product cues, consumers’ quality evaluation is formed by utilising cues or information related to the food products. The supply of sustainably produced high-quality fresh pork products, which is dependent on consumers’ requirements, will support the stance and growth of the South African pork industry. Although literature regarding pork product cues is available, there are limited studies that focus on which intrinsic and extrinsic cues consumers find most relevant in the South African context. Defining these product cues might aid the pork industry in future product development by allowing pork products to match customer desires and, eventually, benefit the consumer by meeting their consumption requirements.

The intrinsic and extrinsic cues of food products influence consumers’ product perception and their perceived value of pork meat. Perceived value plays a significant role in modelling consumers’ purchase intentions and product preferences [27]. Therefore, food producers, industries, and retailers strive to understand how these intrinsic and extrinsic cues impact consumers’ perceived value, hoping to meet consumer expectations and ultimately increase their market share. Literature on the interaction of intrinsic and extrinsic product cues on consumer perception received a lot of attention within a variety of product categories [28]. However, the lack of identified pork cues from literature articles illustrates the need for research on which intrinsic and extrinsic product cues impact consumers’ perceived value of pork meat [29]. Therefore, we hypothesize that the intrinsic and extrinsic product cues influence consumers’ perceived value of pork meat. Furthermore, the pork industry and retailers are unaware of the significant role product cues could play in creating a more optimistic perceived value of pork meat in South Africa. The aim of this paper was to determine the product cues affecting South African consumers’ perceived value of pork meat by conducting a segmentation study on the intrinsic and extrinsic cues.

## 2. Materials and Methods

### 2.1. Sample and Data Collection

The target population for this study was English-literate adult South African meat-eaters currently residing in South Africa and having access to social media. Eating meat is firmly ingrained into the history of South African people [30]. Therefore, it was applicable to obtain information and descriptors on the perceived values South African meat-eaters have for pork meat. This study utilized a quantitative, non-experimental descriptive research design with a cross-sectional survey. A non-probability convenience sampling method was employed to recruit the respondents. A total of 114 adult consumers participated in this study. An online questionnaire was administered using Google Forms version 9.1.5.6 to collect data between February 2024 and April 2024.

### 2.2. Questionnaire

The data collection tool was adapted and formulated from pre-existing standardised questionnaires [31,32,33]. The first section (Section 1) of the questionnaire aimed to determine the socio-demographic profile, i.e., gender, age, employment status, location of home, and dietary classification, including respondents’ frequency of pork meat purchases and consumption, and their perceived value of pork meat. To measure the perceived value of pork meat, participants responded to the question “How do you perceive pork meat products?” In this section, respondents selected the box next to the answer to each question most applicable to their demographics and general information.

Section 2 and Section 3 in the questionnaire adopted a five-point scale to determine the importance of specific intrinsic and extrinsic pork meat cues [31,32,33] to the respondents, ranging from very unimportant (1) to very important (5). The product cues used in the questionnaire were sourced from previous studies [31,32] as these cues have already passed through a four-step exploratory study where the relevance of each cue was determined. Section 2 in the questionnaire focused on the 17 intrinsic cues of pork meat, in which respondents needed to rate the level of importance for each intrinsic cue. However, each cue was rated by selecting the corresponding box. Section 3 followed the same rules and patterns as Section 2; however, Section 3 focused on the 26 extrinsic cues of pork meat.

### 2.3. Data Analysis

Cronbach’s alpha coefficient was used to test the internal consistency of the constructs: (1) pork purchase frequency; (2) perceived value of pork; (3) intrinsic cues; and (4) extrinsic cues. Satisfactory findings were presented for all scales (Cronbach’s alpha > 0.90). The means (of all the scales) were computed and subjected to hierarchical cluster analysis employing Ward’s method using the squared Euclidean distance. A hierarchical cluster analysis was performed on intrinsic and extrinsic cue variables, respectively, to determine the optimal number of clusters. Ward’s method was implemented to group data points into clusters while minimizing the increase in total within-cluster variance. Squared Euclidean distance was used as the similarity measure between cases. Using contrast analyses, the results indicated that each cluster emerged significantly for the two-cluster solution. Robust tests (Welch) were applied to determine the significance of the demographic variables, frequency of pork meat purchases, perceived value of pork meat, intrinsic, and extrinsic cues. Statistical analyses were performed with IBM SPSS Statistics 24.

## 3. Results

### 3.1. Description of the Sample

The majority of the sample were female respondents (71.1%) between the ages of 18 and 44 years (59.9%), working full time (64.8%), living in an urban area (71.4%), and following an omnivorous diet (75%). The sample purchases pork meat mostly on a monthly basis (53.7%) and has a moderate perceived value of pork meat (33.5%). The sample regarded the visual appearance, odour, flavour, and taste of the pork cut as the most important intrinsic cues. Regarding the missing data for the intrinsic cues, eleven respondents’ data were not included, since they did not complete all the items for the intrinsic cues in Section 2 of the online questionnaire. For the extrinsic cues, the sample indicated the packaging, health, quality, additives, origin, and price as important drivers to influence their perceived value of pork meat.

### 3.2. Description of the Clusters

The cluster analyses provided two segments of the intrinsic cues and extrinsic cues affecting South African consumers’ perceived value of pork meat (Table 1). For the intrinsic cues (*n* = 103), the first cluster was described as the sensory seekers (88.3%), and the second cluster was called the indecisive consumers (11.7%)

For the extrinsic cues (*n* = 114), the first cluster was described as the price seekers (51.85%) and the second cluster the preparation inquirers (48.2%).

#### 3.2.1. Intrinsic: The Sensory Seekers

This segment included the highest proportion of female respondents between the ages of 18 and 44 years, working full time and following an omnivorous diet (Table 1). Of all the segments, they mostly purchased pork on a monthly basis and have the highest moderate perceived value of pork meat (Table 2). The sensory seekers regarded the cues, visual appearance, taste, odour, and flavour of pork meat as moderately to very important (Table 3).

#### 3.2.2. Intrinsic: The Indecisive Consumers

This cluster indicated the highest proportion of male respondents who lived in a rural location and who classified themselves as carnivores (Table 1). Interestingly, this segment also contained the highest proportion of flexitarians. This segment purchases pork meat mostly on a monthly basis and has both a neutral and very high perceived value of pork meat, which confirms their indecisive nature (Table 2). The indecisive consumers regarded all the intrinsic cues as very to moderately unimportant (Table 3); however, while the odour cue (1.08) was regarded as less important, the origin cue (1.83) was seen as more important.

#### 3.2.3. Extrinsic: The Price Seekers

This group included a slightly larger proportion of male respondents than the preparation seekers. They included the highest proportion of respondents who were self-employed (Table 1). Of all the clusters, this segment had the highest perceived value of pork meat (Table 2). The price seekers regarded the cues, quality grading, and the price of pork meat as neutral to moderately important (Table 4).

#### 3.2.4. Extrinsic: The Preparation Inquirers

This segment consisted of the highest proportion of respondents working full-time and living in an urban environment (Table 1). This group purchased pork meat products mostly on a weekly basis, and of all the clusters, indicated the highest neutral perceived value of pork meat (Table 2). The preparation inquirers regarded the cues, additives, origin, and packaging as moderately to very important (Table 4).

### 3.3. Intrinsic and Extrinsic Cues

The sensory seekers regarded the type of pork cuts on offer as moderately to very important and indicated that the cues, visual appearance, odour, taste, and flavour are moderately to very important. Interestingly, for the indecisive consumers, the size of the pork meat cut and the origin of the pork meat were slightly more important than the other intrinsic cues; however, the sensory seekers regarded the origin of the pork meat as moderately unimportant to neutral (Table 3).

For the price cue, the price seekers considered the price per kilogram and promotions as neutral to moderately important. Except for the processing cues, the preparation inquirers regarded all the extrinsic cues as moderately to very important. For both segments, the best-before date on the pork product was the most important extrinsic cue. Interestingly, the preparation inquirers indicated the type of packaging (plastic, polystyrene, and skin) as less important extrinsic cues (Table 4).

## 4. Discussion

### 4.1. The Value of Product Cues in Consumers’ Perceived Value of Pork Meat

Cluster analyses provided two consumer segments for the product cues: the sensory seekers and the indecisive consumers for the intrinsic cues, and the price seekers and preparation inquirers for the extrinsic cues.

For the sensory seekers (intrinsic cues), similar results from parallel studies [34,35,36] indicated consumers found the sensory qualities (taste, flavour, appearance, and aroma) of pork meat pleasant. Pork meat’s sensory characteristics are also highly regarded in this study. Consumer’s first interaction with a product is through sight, which instantly predetermines product expectations [37]. Therefore, respondents are motivated by the visual appearance and especially the sensory properties of pork meat, such as the colour of the meat, which influences their purchasing intentions. Positive flavour experiences frequently result in increased satisfaction and repurchase intention from consumers. Whereby consumers perceive good taste and flavour with good quality, prompting them to pay more for pork meat products [38]. Therefore, it is clear that the expected organoleptic sensation of flavour influences consumers’ perceived value of pork meat. Consumers are more likely to attach positive sensory experiences to a food product’s taste when they have a positive view of the product, which strengthens their acceptance of the overall product [39]. This emphasizes the importance of taste within consumers’ purchasing intentions and perceived value of pork meat. Therefore, previous studies [40,41,42,43] and this study confirm that sensory characteristics are key influences in forming consumers’ perceived value of pork meat. Studies have found that sensory characteristics influence consumers to conjure emotions and attitudes that affect their perceived value [44,45]. As these intrinsic cues have a direct impact on consumers’ satisfaction, taste and flavour are instantaneous sensory sensations that can influence how pleasant the pork meat is during consumption. A pleasant consumer experience increases overall satisfaction, which increases the consumer’s motivation to have a higher perceived value for the product [38].

Regarding the indecisive segment (intrinsic cues), indecisiveness is defined as “difficulty in making all sorts of life decisions, whether they are of great or little significance.” When indecisive consumers face a decision task, they delay making a decision because they cannot make up their minds [46]. Findings indicate that indecisive individuals may be unable to efficiently organize stimuli in their environment, resulting in over-structuring or over-categorizing of information. Because this additional categorizing and structuring takes time and effort, it may lead to increased decision latency, which is consistent with the finding that individuals who are highly indecisive require more time to make decisions [47]. Indecisiveness may make it difficult to establish what intrinsic cues are important when choosing pork meat products and may also negatively influence consumers’ perceived value of pork meat products.

Extrinsic cues function as indicators for consumers to deduce product quality of unfamiliar items, brands, or products when there is an inaccessible absence of intrinsic product cues [48]. Research has indicated that consumers utilise extrinsic quality cues to drive their purchasing intentions. Implying that any visual (quality assurance ratings) and tactile (meat grading) aspect of the product represents quality cues capable of influencing consumers’ perceived value [49].

The price seekers (extrinsic cues) value the importance of the price of pork meat products. Previous studies confirmed that price is the most important extrinsic cue in the formulation of consumers’ perceived value and the most influential in consumers’ purchasing intention [50]. A product’s price serves as a salient extrinsic cue that conveys information about the product’s quality and perceived value. Consumers use price as a quality indicator prior to product usage [51]. Therefore, price, an economic aspect, is an important contributor to consumers’ perceived value when purchasing pork meat products.

The price seekers also valued the quality of pork meat products as an important food cue. Product quality influences consumer satisfaction, which can enhance or reduce their overall perceived value of that product [52]. Therefore, the quality of food products is the foremost reason for a consumer’s purchasing intention. Thus, food manufacturers promote certificates of credibility (e.g., Pork 360 certified manufacturers) which emphasise their product quality attributes to invoke a higher perceived value [53].

For the preparation inquirers, it is evident that the preparation (quality, additives, origin, and packaging) of pork meat is an important extrinsic contributor in shaping local consumers’ perceived value of pork meat. Consumers view these extrinsic cues as quality indicators. Regarding the packaging cues, the best-before date on pork meat packaging and vacuum-pack packaging are considered important quality indicators. According to prior research, cues from packaging material influence consumers’ perceived value significantly [54]. Furthermore, [55] confirmed that packaging exerts a persistent influence on product-level expectations and is perceived positively when it improves consumers’ view of the product’s flavour and quality. A clear and visible best-before date will assist the consumer in determining the freshness and sensory qualities of the pork meat products [56], which illustrates the importance of the best-before date on pork meat packaging. Therefore, packaging with unblemished labelling conveys a sense of care and attention to detail, which emphasizes superior production quality [55].

When considering the additive cues, the absence of artificial additives had the most influence on consumers’ perceived value. Considering that consumer acceptance of additives in meat processing is a key obstacle for food processing industries due to food safety demands from consumers [57]. It is evident that the absence of additives provides consumers with more control over their own products and what goes into their bodies, positively impacting their perceived value. At the same time, the absence of additives and preservatives grows within the increasing popularity of natural and unprocessed meals [58]. These perspectives foster the perceived value of pork meat as a healthier alternative.

### 4.2. Consumer Implications

The segmentation study indicates the similarities and differences between the clusters and suggests the development of a specific product cue lexicon for the South African pork market to influence consumers’ perceived value of pork meat. The sensory seekers and preparation inquirers indicate that consumers value the eating experience and quality of pork meat. Combining an enhanced eating experience with the addition and awareness of preparation factors would help consumers improve their quality evaluations of pork meat products within the South African context. Evidently, the indecisive consumers (intrinsic) and the price seekers (extrinsic) need to be targeted, as we suspect that these consumer segments will escalate, which could have a limited effect on consumers’ perceived value of pork meat. Therefore, emphasising more on the sensory and preparation characteristics may improve consumer satisfaction and modify their perceived value positively, thus increasing market demand.

### 4.3. Managerial Implications

This information may assist manufacturers and marketers involved in the pork industry in emphasising crucial cues that are consistent with consumer preferences, such as the absence of additives, effective packaging, transparent pricing, origin, and traceability. These product cues could aid the South African pork industry through consumer-directed product development, which utilises the defined intrinsic and extrinsic cues that meet consumers’ requirements, while the pork industry stakeholders reap the benefits from increased consumer satisfaction. Enhancing these cues allows pork manufacturing brands to generate positive sensory experiences that encourage consumer loyalty, justify higher price points, and have a substantial impact on consumers’ perceived value, which in turn, generates a supporting framework for industrial growth.

This understanding pushes manufacturers and marketers to improve the product cues identified in order to better match consumer expectations according to different consumer segments. Implying that marketers would need to emphasise the promotion of pork meat’s sensory characteristics alongside its preparation while acknowledging that other consumer segments, i.e., indecisive consumers (intrinsic) and the price seekers (extrinsic) should start to play a more important role in establishing an optimistic perceived value of pork-derived meat products within consumers.

This study provides a new perspective and approach for research on product cues as the identified cues could: (1) help South Africa and the relevant stakeholders benefit by providing sought-after pork products for the masses alongside extending the research community’s understanding of pork cues; (2) inform the pork industry about which attributes to accentuate in their product development to comply with consumer requirements; and (3) serve as distinct qualities of pork meat—such as its sensory and preparation characteristics—meaning that a more detailed investigation of how these cues particularly affect consumers’ perceived value of pork meat is required.

### 4.4. Recommendations and Limitations

The use of quantitative methods may force researchers to simplify complex phenomena into numerical data, therefore limiting our study to elaborate on consumers’ simplified views and perceptions of the intrinsic and extrinsic cues affecting their perceived value of pork meat. Therefore, further studies are required to employ qualitative research methodologies to fully explore and grasp how intrinsic and extrinsic cues affect consumers’ perceived value of pork meat.

The results of this study could not be generalised to the entire South African population, which limits the generalisability of the findings across diverse groups. A larger sample size using different consumer groups is therefore recommended for future research on pork meat cues.

We recommended that forthcoming research could investigate the deeper understanding of consumers’ perceived value of pork meat through consumer behaviour studies to comprehend how pork product cues affect consumers’ perceived values. Furthermore, the findings demonstrated the importance of specific product cues within consumers’ perceived value of pork meat.

## 5. Conclusions

Our study’s findings provide important insights into how consumers assess pork meat, emphasising the intrinsic and extrinsic cues that are valued by consumers. Findings from this study confirmed consumers’ positive view towards pork meat and emphasised the importance of product cues enabling the pork industry to focus more on the sensory and preparation characteristics of pork meat, not only to improve the quality of products but also to enhance consumers’ overall eating experience to match their expectations. Ultimately, consumers could benefit from the cue-adjusted pork meat products that will influence their perceived value of this affordable and versatile food. Consequently, it is advised that subsequent research should focus on consumer evaluation and satisfaction with sensory and preparation characteristics, with emphasis on sensory descriptors of pork meat and how manufacturers can contribute to communicating these characteristics prior to consumer consumption, with the objective of elevating perceived value.

## Figures and Tables

**Table 1:** Socio-demographic features by clusters for intrinsic and extrinsic cues.

	Intrinsic (*n* = 103) Sensory Seekers *n* = 91 88.3%	Indecisive Consumers *n* = 12 11.7%	Extrinsic (*n* = 114) Price Seekers *n* = 59 51.8%	Preparation Inquirers *n* = 55 48.2%
Gender				
Male	20.9%	45.5%	27.6%	21.8%
Female	79.1%	54.5%	72.4%	78.2%
Age				
18–44	72.5%	33.3%	62.7%	70.9%
45–64	19.8%	33.3%	28.8%	16.4%
≥65	7.7%	33.3%	8.5%	12.7%
Employment status				
Full time	70.9%	50.0%	66.1%	72.0%
Pensioner	5.8%	20.0%	5.3%	10.0%
Self employed	12.8%	10.0%	16.1%	10.0%
Student	10.5%	20.0%	12.5%	8.0%
Location of home				
Urban	78.0%	58.3%	71.2%	78.2%
Rural	22.0%	41.7%	28.8%	21.8%
Dietary classification ^1^*^2^**				
Carnivore	0%	8.3%	1.1%	0%
Flexitarian	11.0%	25.0%	6.6%	12.7%
Omnivore	89.0%	66.7%	57.1%	87.3%

Note. * *p* < 0.05; ** *p* < 0.01; ^1^ = Intrinsic; ^2^ = Extrinsic.

**Table 2:** Pork purchase frequency and perceived value of pork meat by clusters.

	Intrinsic (*n* = 103) Sensory Seekers *n* = 91 88.3%	Indecisive Consumers *n* = 12 11.7%	Extrinsic (*n* = 114) Price Seekers *n* = 59 51.8%	Preparation Inquirer *n* = 55 48.2%
Pork purchase frequency ^1^**^2^***				
Daily	0%	16.7%	3.4%	0%
Weekly	34%	25.0%	28.8%	38.2%
Monthly	63.8%	50.0%	42.9%	58.2%
Quarterly	2.2%	8.3%	1.7%	3.6%
Perceived value of pork				
Neutral	34.0%	33.3%	33.9%	38.2%
Moderate	38.5%	25.0%	33.9%	36.4%
High	11.0%	8.4%	11.9%	9.1%
Very high	16.5%	33.3%	20.3%	16.4%

Note. ** *p* < 0.01; *** *p* < 0.001; ^1^ = Intrinsic; ^2^ = Extrinsic.

**Table 3:** Mean scores reflecting the importance of intrinsic cues [24] for pork meat as evaluated by two clusters of respondents (*n* = 103).

	Sensory Seekers *n* = 91 88.3%	Indecisive Consumers *n* = 12 11.7%
Physical attributes	3.72	1.60
The type of pork cuts on offer (e.g., Loin ribs vs. Belly ribs)	4.04	1.58
The thickness of the cut	3.91	1.58
The size of the cut	3.97	1.83
The weight of the pork cut (Kg) **	3.42	1.67
The nutritional composition of pork **	3.24	1.33
Visual appearance	4.13	1.34
The colour of the meat *	4.62	1.17
The colour of the fat ***	4.30	1.17
The fat on the pork cut has been trimmed **	3.80	1.67
The wetness of the pork cut (e.g., water resembling liquid on the surface of the pork cut) *	3.78	1.33
Odour	4.42	1.08
No smell or taste of boar taint (boar odour during cooking or taste during consumption) ***	4.42	1.08
Texture	3.83	1.42
The leanness of pork cuts (low in fat) **	3.62	1.67
The firmness of the pork cut *	4.03	1.17
Taste	4.14	1.38
The marbling in pork cuts (the amount of intermuscular fat content) *	3.49	1.58
The taste of the pork cut	4.79	1.17
Flavour	4.53	1,25
The succulentness of the pork cut (e.g., tenderness and juiciness) *	4.48	1.25
The flavour of the pork cut*	4.57	1.25
Origin	2.20	1.83
The type of pig breed (Landrace, Large White & Duroc) *	2.20	1.83

Note. * *p* < 0.05; ** *p* < 0.01; *** *p* < 0.001. Mean scores on scales: 1 = Very unimportant; 2 = Moderately unimportant; 3 = Neutral; 4 = Moderately important; 5 = Very important.

**Table 4:** Mean scores reflecting the importance of extrinsic cues [24] for pork meat as evaluated by two clusters of respondents (*n* = 114).

	Price Seekers *n* = 59 51.8%	Preparation Inquirers *n* = 55 48.2%
Quality	2.93	4.15
Quality grading of pork cuts (Grade A, B& C cuts) *	3.14	4.11
Quality assurance ratings (e.g., Pork 360 certified)	2.71	4.18
Additives	2.71	4.19
No preservatives (salt, BHA, BHT, nitrites, citric acid) *	2.59	4.13
Absence of artificial additives (flavours or colourants) ***	2.83	4.24
Origin	2.64	4.26
Branded pork meat products (e.g., Eskort vs. Pick ‘n Pay Brand) **	2.86	4.36
Slaughter date of the pork	2.85	4.02
Pork product traceability information (where and when the product was produced) **	2.36	4.22
Raised and slaughtered with high levels of animal welfare (stress free environments) **	2.46	4.29
Sustainably farmed and produced pork products **	2.69	4.42
Price	3.39	4.47
Price per kilogram ***	3.46	4.60
Promotions (e.g., discounts or sales) ***	3.31	4.33
Processing	2.91	3.94
Chilled pork products (refrigerated products) ***	3.56	4.47
Frozen pork products (freezer products)	2.53	3.98
Pork products infused with moisture (e.g., salt brine injected pork shoulder)	2.63	3.36
Packaging	2.86	4.10
Plastic packaging (products packed in plastic bags)	2.71	3.49
Polystyrene packaging (butchery type packaging, polystyrene bottom with glad wrap plastic) *	2.68	3.78
Skin packaging (black plastic tray with clear view plastic on top) *	2.86	3.93
Vacuum-pack packaging ***	3.12	4.33
Best before date on the pork product ***	3.80	4.95
Nutritional information on the packaging	2.51	4.24
Cooking and serving suggestions on the packaging	2.34	3.75
Health	2.73	4.13
Visible health star ratings (rating of the overall nutritional profile of the packaged good) **	2.73	4.13
Feed & GMOs	2.20	4.01
Hormone Growth Promotant Free (animals free from growth hormones)	2.51	4.22
No Genetically Modified Organism (animal did not undergo genetic modifications)	2.41	4.11
Type of animal feed—organic feed (grass or grain)	1.97	3.96
Type of animal feed—EPOL Feed pellets, grass fed, grain fed	1.92	3.76

Note. * *p* < 0.05; ** *p* < 0.01; *** *p* < 0.001. Mean scores on scales: 1 = Very unimportant; 2 = Moderately unimportant; 3 = Neutral; 4 = Moderately important; 5 = Very important.

## Data Availability

The original contributions presented in the study are included in this article; further inquiries can be directed to the corresponding author.
